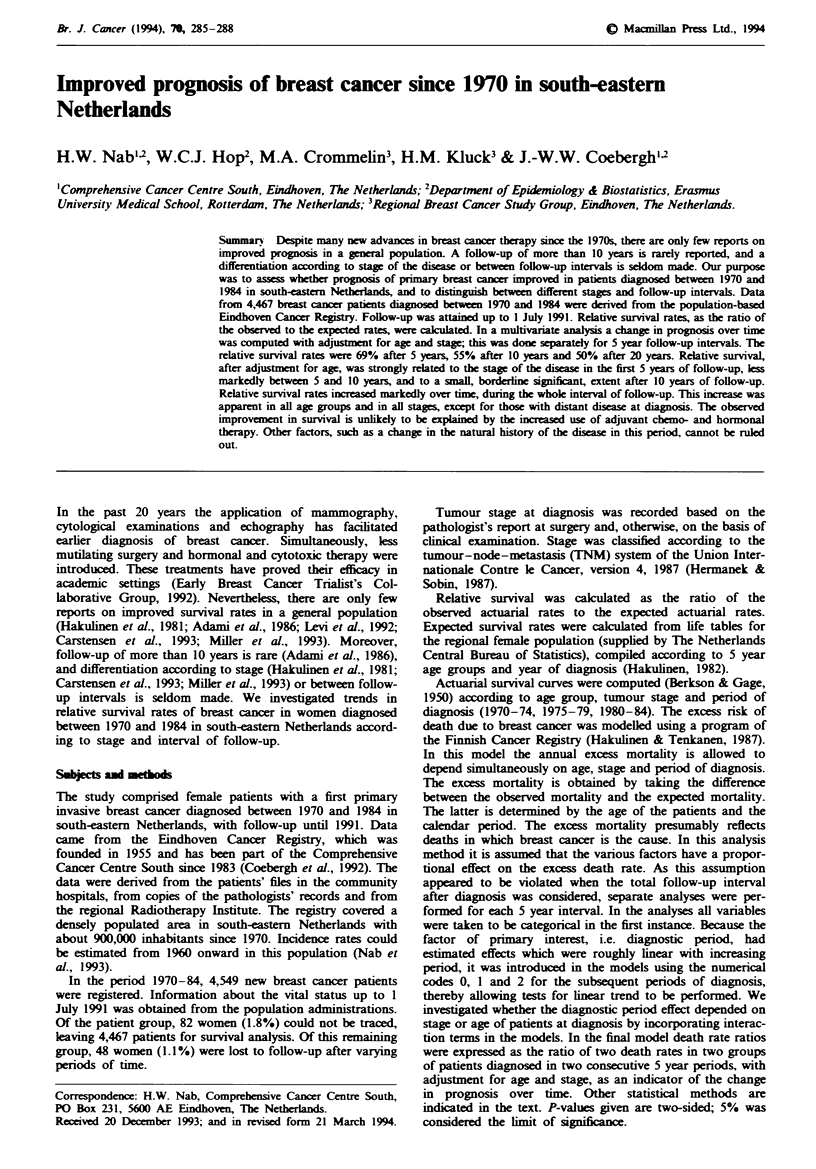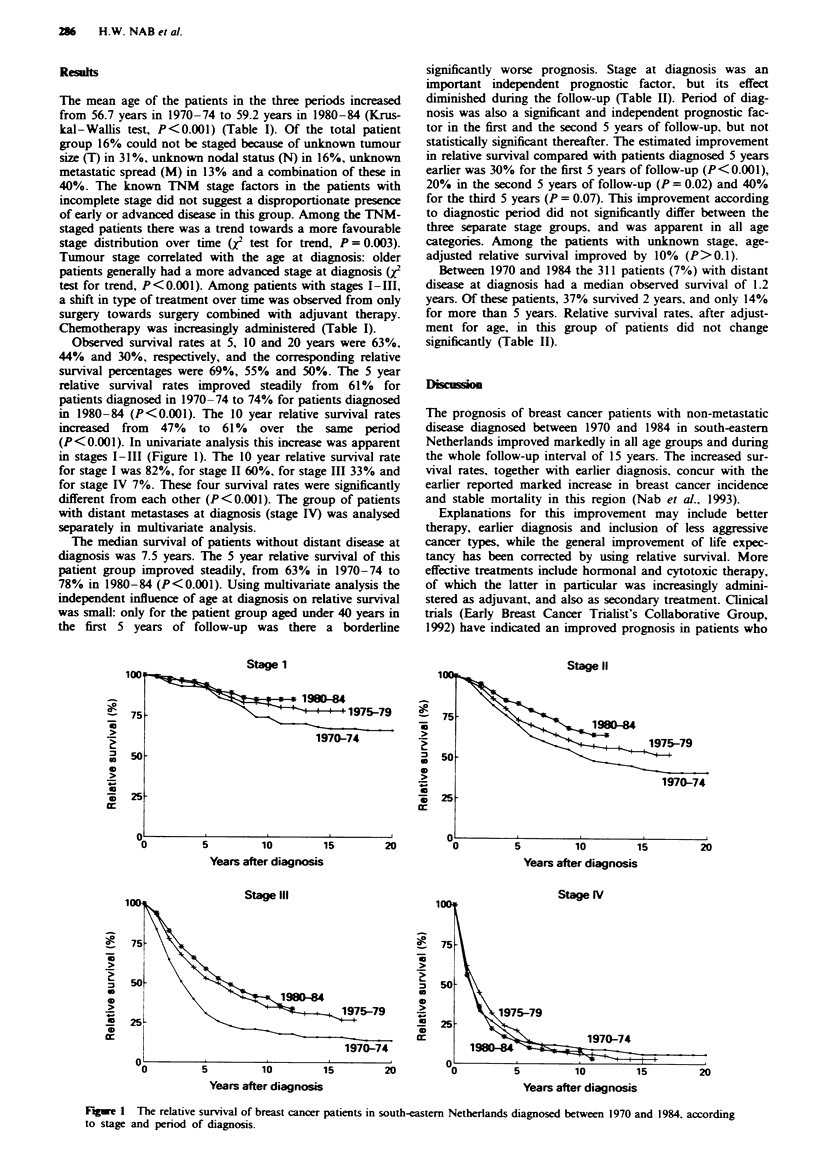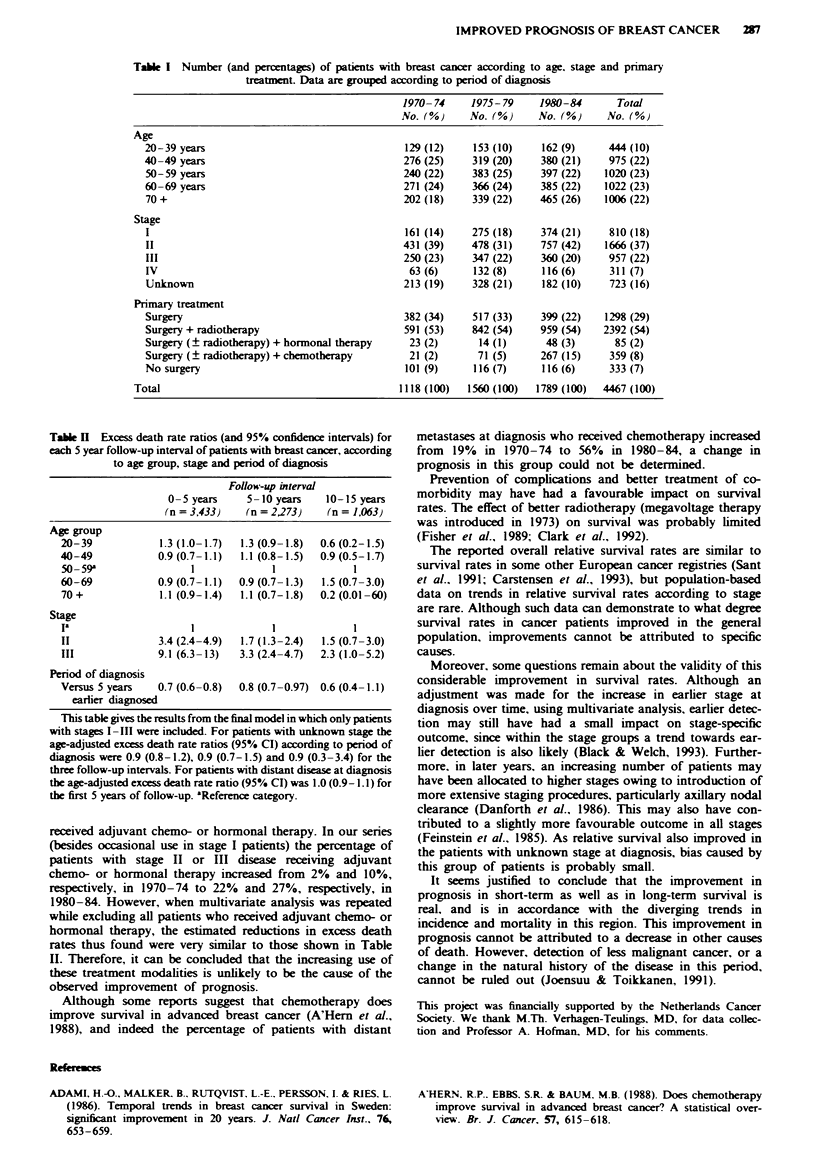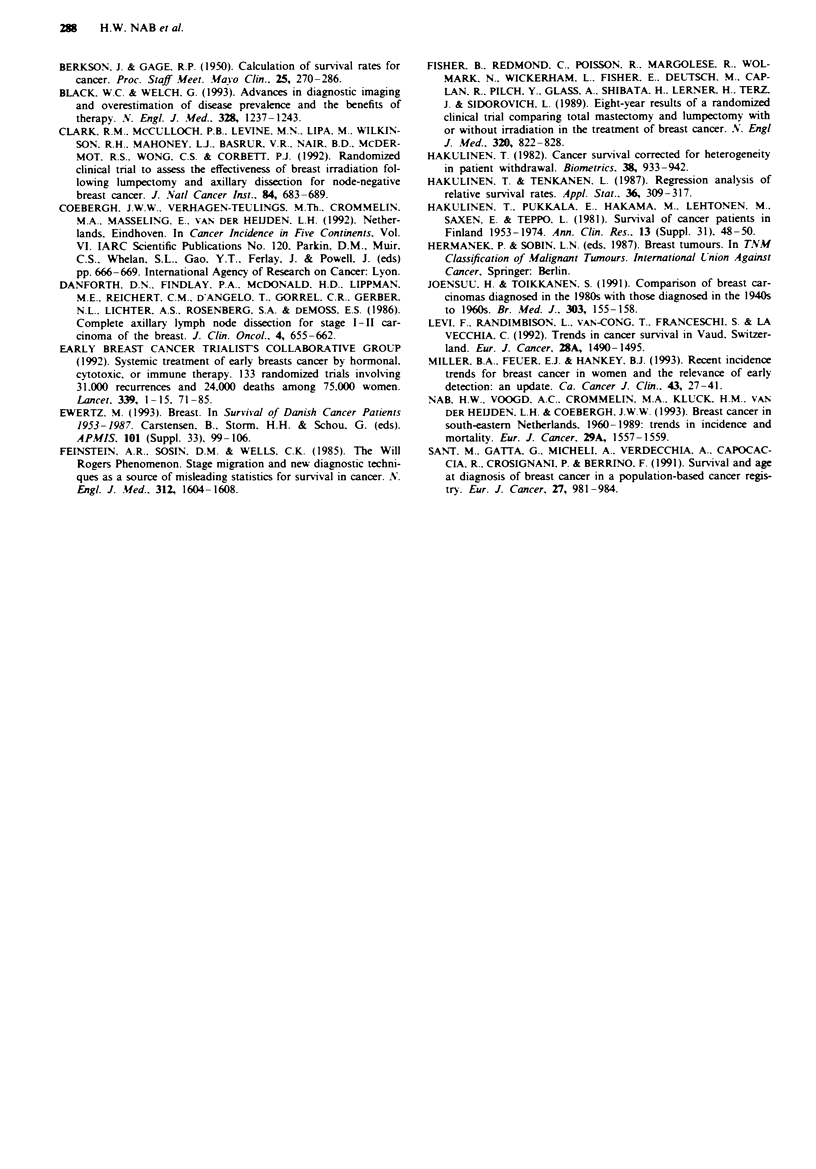# Improved prognosis of breast cancer since 1970 in south-eastern Netherlands.

**DOI:** 10.1038/bjc.1994.293

**Published:** 1994-08

**Authors:** H. W. Nab, W. C. Hop, M. A. Crommelin, H. M. Kluck, J. W. Coebergh

**Affiliations:** Comprehensive Cancer Centre South, Eindhoven, The Netherlands.

## Abstract

Despite many new advances in breast cancer therapy since the 1970s, there are only few reports on improved prognosis in a general population. A follow-up of more than 10 years is rarely reported, and a differentiation according to stage of the disease or between follow-up intervals is seldom made. Our purpose was to assess whether prognosis of primary breast cancer improved in patients diagnosed between 1970 and 1984 in south-eastern Netherlands, and to distinguish between different stages and follow-up intervals. Data from 4,467 breast cancer patients diagnosed between 1970 and 1984 were derived from the population-based Eindhoven Cancer Registry. Follow-up was attained up to 1 July 1991. Relative survival rates, as the ratio of the observed to the expected rates, were calculated. In a multivariate analysis a change in prognosis over time was computed with adjustment for age and stage; this was done separately for 5 year follow-up intervals. The relative survival rates were 69% after 5 years, 55% after 10 years and 50% after 20 years. Relative survival, after adjustment for age, was strongly related to the stage of the disease in the first 5 years of follow-up, less markedly between 5 and 10 years, and to a small, borderline significant, extent after 10 years of follow-up. Relative survival rates increased markedly over time, during the whole interval of follow-up. This increase was apparent in all age groups and in all stages, except for those with distant disease at diagnosis. The observed improvement in survival is unlikely to be explained by the increased use of adjuvant chemo- and hormonal therapy. Other factors, such as a change in the natural history of the disease in this period, cannot be ruled out.


Br. J. Cancer (1994), 70, 285-288 © Macmillan Press Ltd., 1994

### Improved prognosis of breast cancer since 1970 in south-eastern Netherlands

H.W. Nab[1,2], W.C.J. Hop[2], M.A. Crommelin[3], H.M. Kluck[3] & J.-W.W. Coebergh[1,2]

[1]*Comprehensive Cancer Centre South, Eindhoven, The Netherlands;* [2]*Department of Epidemiology & Biostatistics, Erasmus University Medical School, Rotterdam, The Netherlands;* [3]*Regional Breast Cancer Study Group, Eindhoven, The Netherlands.*

**Summary** Despite many new advances in breast cancer therapy since the 1970s, there are only few reports on improved prognosis in a general population. A follow-up of more than 10 years is rarely reported, and a differentiation according to stage of the disease or between follow-up intervals is seldom made. Our purpose was to assess whether prognosis of primary breast cancer improved in patients diagnosed between 1970 and 1984 in south-eastern Netherlands, and to distinguish between different stages and follow-up intervals. Data from 4,467 breast cancer patients diagnosed between 1970 and 1984 were derived from the population-based Eindhoven Cancer Registry. Follow-up was attained up to 1 July 1991. Relative survival rates, as the ratio of the observed to the expected rates, were calculated. In a multivariate analysis a change in prognosis over time was computed with adjustment for age and stage; this was done separately for 5 year follow-up intervals. The relative survival rates were 69% after 5 years, 55% after 10 years and 50% after 20 years. Relative survival, after adjustment for age, was strongly related to the stage of the disease in the first 5 years of follow-up, less markedly between 5 and 10 years, and to a small, borderline significant, extent after 10 years of follow-up. Relative survival rates increased markedly over time, during the whole interval of follow-up. This increase was apparent in all age groups and in all stages, except for those with distant disease at diagnosis. The observed improvement in survival is unlikely to be explained by the increased use of adjuvant chemo- and hormonal therapy. Other factors, such as a change in the natural history of the disease in this period, cannot be ruled out.

In the past 20 years the application of mammography, cytological examinations and echography has facilitated earlier diagnosis of breast cancer. Simultaneously, less mutilating surgery and hormonal and cytotoxic therapy were introduced. These treatments have proved their efficacy in academic settings (Early Breast Cancer Trialist's Collaborative Group, 1992). Nevertheless, there are only few reports on improved survival rates in a general population (Hakulinen *et al.*, 1981; Adami *et al.*, 1986; Levi *et al.*, 1992; Carstensen *et al.*, 1993; Miller *et al.*, 1993). Moreover, follow-up of more than 10 years is rare (Adami *et al.*, 1986), and differentiation according to stage (Hakulinen *et al.*, 1981; Carstensen *et al.*, 1993; Miller *et al.*, 1993) or between follow-up intervals is seldom made. We investigated trends in relative survival rates of breast cancer in women diagnosed between 1970 and 1984 in south-eastern Netherlands according to stage and interval of follow-up.

### Subjects and methods

The study comprised female patients with a first primary invasive breast cancer diagnosed between 1970 and 1984 in south-eastern Netherlands, with follow-up until 1991. Data came from the Eindhoven Cancer Registry, which was founded in 1955 and has been part of the Comprehensive Cancer Centre South since 1983 (Coebergh *et al.*, 1992). The data were derived from the patients' files in the community hospitals, from copies of the pathologists' records and from the regional Radiotherapy Institute. The registry covered a densely populated area in south-eastern Netherlands with about 900,000 inhabitants since 1970. Incidence rates could be estimated from 1960 onward in this population (Nab *et al.*, 1993).

In the period 1970-84, 4,549 new breast cancer patients were registered. Information about the vital status up to 1 July 1991 was obtained from the population administrations. Of the patient group, 82 women (1.8%) could not be traced, leaving 4,467 patients for survival analysis. Of this remaining group, 48 women (1.1%) were lost to follow-up after varying periods of time.

Correspondence: H.W. Nab, Comprehensive Cancer Centre South, PO Box 231, 5600 AE Eindhoven, The Netherlands.
Received 20 December 1993; and in revised form 21 March 1994.

Tumour stage at diagnosis was recorded based on the pathologist's report at surgery and, otherwise, on the basis of clinical examination. Stage was classified according to the tumour-node-metastasis (TNM) system of the Union Internationale Contre le Cancer, version 4, 1987 (Hermanek & Sobin, 1987).

Relative survival was calculated as the ratio of the observed actuarial rates to the expected actuarial rates. Expected survival rates were calculated from life tables for the regional female population (supplied by The Netherlands Central Bureau of Statistics), compiled according to 5 year age groups and year of diagnosis (Hakulinen, 1982).

Actuarial survival curves were computed (Berkson & Gage, 1950) according to age group, tumour stage and period of diagnosis (1970-74, 1975-79, 1980-84). The excess risk of death due to breast cancer was modelled using a program of the Finnish Cancer Registry (Hakulinen & Tenkanen, 1987). In this model the annual excess mortality is allowed to depend simultaneously on age, stage and period of diagnosis. The excess mortality is obtained by taking the difference between the observed mortality and the expected mortality. The latter is determined by the age of the patients and the calendar period. The excess mortality presumably reflects deaths in which breast cancer is the cause. In this analysis method it is assumed that the various factors have a proportional effect on the excess death rate. As this assumption appeared to be violated when the total follow-up interval after diagnosis was considered, separate analyses were performed for each 5 year interval. In the analyses all variables were taken to be categorical in the first instance. Because the factor of primary interest, i.e. diagnostic period, had estimated effects which were roughly linear with increasing period, it was introduced in the models using the numerical codes 0, 1 and 2 for the subsequent periods of diagnosis, thereby allowing tests for linear trend to be performed. We investigated whether the diagnostic period effect depended on stage or age of patients at diagnosis by incorporating interaction terms in the models. In the final model death rate ratios were expressed as the ratio of two death rates in two groups of patients diagnosed in two consecutive 5 year periods, with adjustment for age and stage, as an indicator of the change in prognosis over time. Other statistical methods are indicated in the text. *P*-values given are two-sided; 5% was considered the limit of significance.

### Results

The mean age of the patients in the three periods increased from 56.7 years in 1970–74 to 59.2 years in 1980–84 (Kruskal–Wallis test, $P<0.001$) (Table I). Of the total patient group 16% could not be staged because of unknown tumour size (T) in 31%, unknown nodal status (N) in 16%, unknown metastatic spread (M) in 13% and a combination of these in 40%. The known TNM stage factors in the patients with incomplete stage did not suggest a disproportionate presence of early or advanced disease in this group. Among the TNM-staged patients there was a trend towards a more favourable stage distribution over time ($\chi^2$ test for trend, $P=0.003$). Tumour stage correlated with the age at diagnosis: older patients generally had a more advanced stage at diagnosis ($\chi^2$ test for trend, $P<0.001$). Among patients with stages I–III, a shift in type of treatment over time was observed from only surgery towards surgery combined with adjuvant therapy. Chemotherapy was increasingly administered (Table I).

Observed survival rates at 5, 10 and 20 years were 63%, 44% and 30%, respectively, and the corresponding relative survival percentages were 69%, 55% and 50%. The 5 year relative survival rates improved steadily from 61% for patients diagnosed in 1970–74 to 74% for patients diagnosed in 1980–84 ($P<0.001$). The 10 year relative survival rates increased from 47% to 61% over the same period ($P<0.001$). In univariate analysis this increase was apparent in stages I–III (Figure 1). The 10 year relative survival rate for stage I was 82%, for stage II 60%, for stage III 33% and for stage IV 7%. These four survival rates were significantly different from each other ($P<0.001$). The group of patients with distant metastases at diagnosis (stage IV) was analysed separately in multivariate analysis.

The median survival of patients without distant disease at diagnosis was 7.5 years. The 5 year relative survival of this patient group improved steadily, from 63% in 1970–74 to 78% in 1980–84 ($P<0.001$). Using multivariate analysis the independent influence of age at diagnosis on relative survival was small: only for the patient group aged under 40 years in the first 5 years of follow-up was there a borderline significantly worse prognosis. Stage at diagnosis was an important independent prognostic factor, but its effect diminished during the follow-up (Table II). Period of diagnosis was also a significant and independent prognostic factor in the first and the second 5 years of follow-up, but not statistically significant thereafter. The estimated improvement in relative survival compared with patients diagnosed 5 years earlier was 30% for the first 5 years of follow-up ($P<0.001$), 20% in the second 5 years of follow-up ($P=0.02$) and 40% for the third 5 years ($P=0.07$). This improvement according to diagnostic period did not significantly differ between the three separate stage groups, and was apparent in all age categories. Among the patients with unknown stage, age-adjusted relative survival improved by 10% ($P>0.1$).

Between 1970 and 1984 the 311 patients (7%) with distant disease at diagnosis had a median observed survival of 1.2 years. Of these patients, 37% survived 2 years, and only 14% for more than 5 years. Relative survival rates, after adjustment for age, in this group of patients did not change significantly (Table II).

### Discussion

The prognosis of breast cancer patients with non-metastatic disease diagnosed between 1970 and 1984 in south-eastern Netherlands improved markedly in all age groups and during the whole follow-up interval of 15 years. The increased survival rates, together with earlier diagnosis, concur with the earlier reported marked increase in breast cancer incidence and stable mortality in this region (Nab *et al.*, 1993).

Explanations for this improvement may include better therapy, earlier diagnosis and inclusion of less aggressive cancer types, while the general improvement of life expectancy has been corrected by using relative survival. More effective treatments include hormonal and cytotoxic therapy, of which the latter in particular was increasingly administered as adjuvant, and also as secondary treatment. Clinical trials (Early Breast Cancer Trialist's Collaborative Group, 1992) have indicated an improved prognosis in patients who

**Figure 1** The relative survival of breast cancer patients in south-eastern Netherlands diagnosed between 1970 and 1984, according to stage and period of diagnosis.

**Table I** Number (and percentages) of patients with breast cancer according to age, stage and primary treatment. Data are grouped according to period of diagnosis

| | *1970-74* No. (%) | *1975-79* No. (%) | *1980-84* No. (%) | *Total* No. (%) |
|---|---|---|---|---|
| Age | | | | |
| 20-39 years | 129 (12) | 153 (10) | 162 (9) | 444 (10) |
| 40-49 years | 276 (25) | 319 (20) | 380 (21) | 975 (22) |
| 50-59 years | 240 (22) | 383 (25) | 397 (22) | 1020 (23) |
| 60-69 years | 271 (24) | 366 (24) | 385 (22) | 1022 (23) |
| 70 + | 202 (18) | 339 (22) | 465 (26) | 1006 (22) |
| Stage | | | | |
| I | 161 (14) | 275 (18) | 374 (21) | 810 (18) |
| II | 431 (39) | 478 (31) | 757 (42) | 1666 (37) |
| III | 250 (23) | 347 (22) | 360 (20) | 957 (22) |
| IV | 63 (6) | 132 (8) | 116 (6) | 311 (7) |
| Unknown | 213 (19) | 328 (21) | 182 (10) | 723 (16) |
| Primary treatment | | | | |
| Surgery | 382 (34) | 517 (33) | 399 (22) | 1298 (29) |
| Surgery + radiotherapy | 591 (53) | 842 (54) | 959 (54) | 2392 (54) |
| Surgery (± radiotherapy) + hormonal therapy | 23 (2) | 14 (1) | 48 (3) | 85 (2) |
| Surgery (± radiotherapy) + chemotherapy | 21 (2) | 71 (5) | 267 (15) | 359 (8) |
| No surgery | 101 (9) | 116 (7) | 116 (6) | 333 (7) |
| Total | 1118 (100) | 1560 (100) | 1789 (100) | 4467 (100) |

**Table II** Excess death rate ratios (and 95% confidence intervals) for each 5 year follow-up interval of patients with breast cancer, according to age group, stage and period of diagnosis

| | *Follow-up interval* 0-5 years (n = *3,433*) | 5-10 years (n = *2,273*) | 10-15 years (n = *1,063*) |
|---|---|---|---|
| Age group | | | |
| 20-39 | 1.3 (1.0-1.7) | 1.3 (0.9-1.8) | 0.6 (0.2-1.5) |
| 40-49 | 0.9 (0.7-1.1) | 1.1 (0.8-1.5) | 0.9 (0.5-1.7) |
| 50-59[a] | 1 | 1 | 1 |
| 60-69 | 0.9 (0.7-1.1) | 0.9 (0.7-1.3) | 1.5 (0.7-3.0) |
| 70 + | 1.1 (0.9-1.4) | 1.1 (0.7-1.8) | 0.2 (0.01-60) |
| Stage | | | |
| I[a] | 1 | 1 | 1 |
| II | 3.4 (2.4-4.9) | 1.7 (1.3-2.4) | 1.5 (0.7-3.0) |
| III | 9.1 (6.3-13) | 3.3 (2.4-4.7) | 2.3 (1.0-5.2) |
| Period of diagnosis | | | |
| Versus 5 years earlier diagnosed | 0.7 (0.6-0.8) | 0.8 (0.7-0.97) | 0.6 (0.4-1.1) |

This table gives the results from the final model in which only patients with stages I-III were included. For patients with unknown stage the age-adjusted excess death rate ratios (95% CI) according to period of diagnosis were 0.9 (0.8-1.2), 0.9 (0.7-1.5) and 0.9 (0.3-3.4) for the three follow-up intervals. For patients with distant disease at diagnosis the age-adjusted excess death rate ratio (95% CI) was 1.0 (0.9-1.1) for the first 5 years of follow-up. [a]Reference category.

received adjuvant chemo- or hormonal therapy. In our series (besides occasional use in stage I patients) the percentage of patients with stage II or III disease receiving adjuvant chemo- or hormonal therapy increased from 2% and 10%, respectively, in 1970-74 to 22% and 27%, respectively, in 1980-84. However, when multivariate analysis was repeated while excluding all patients who received adjuvant chemo- or hormonal therapy, the estimated reductions in excess death rates thus found were very similar to those shown in Table II. Therefore, it can be concluded that the increasing use of these treatment modalities is unlikely to be the cause of the observed improvement of prognosis.

Although some reports suggest that chemotherapy does improve survival in advanced breast cancer (A'Hern *et al.*, 1988), and indeed the percentage of patients with distant metastases at diagnosis who received chemotherapy increased from 19% in 1970-74 to 56% in 1980-84, a change in prognosis in this group could not be determined.

Prevention of complications and better treatment of co-morbidity may have had a favourable impact on survival rates. The effect of better radiotherapy (megavoltage therapy was introduced in 1973) on survival was probably limited (Fisher *et al.*, 1989; Clark *et al.*, 1992).

The reported overall relative survival rates are similar to survival rates in some other European cancer registries (Sant *et al.*, 1991; Carstensen *et al.*, 1993), but population-based data on trends in relative survival rates according to stage are rare. Although such data can demonstrate to what degree survival rates in cancer patients improved in the general population, improvements cannot be attributed to specific causes.

Moreover, some questions remain about the validity of this considerable improvement in survival rates. Although an adjustment was made for the increase in earlier stage at diagnosis over time, using multivariate analysis, earlier detection may still have had a small impact on stage-specific outcome, since within the stage groups a trend towards earlier detection is also likely (Black & Welch, 1993). Furthermore, in later years, an increasing number of patients may have been allocated to higher stages owing to introduction of more extensive staging procedures, particularly axillary nodal clearance (Danforth *et al.*, 1986). This may also have contributed to a slightly more favourable outcome in all stages (Feinstein *et al.*, 1985). As relative survival also improved in the patients with unknown stage at diagnosis, bias caused by this group of patients is probably small.

It seems justified to conclude that the improvement in prognosis in short-term as well as in long-term survival is real, and is in accordance with the diverging trends in incidence and mortality in this region. This improvement in prognosis cannot be attributed to a decrease in other causes of death. However, detection of less malignant cancer, or a change in the natural history of the disease in this period, cannot be ruled out (Joensuu & Toikkanen, 1991).

This project was financially supported by the Netherlands Cancer Society. We thank M.Th. Verhagen-Teulings, MD, for data collection and Professor A. Hofman, MD, for his comments.

### References

ADAMI, H.-O., MALKER, B., RUTQVIST, L.-E., PERSSON, I. & RIES, L. (1986). Temporal trends in breast cancer survival in Sweden: significant improvement in 20 years. *J. Natl Cancer Inst.*, **76**, 653-659.

A'HERN, R.P., EBBS, S.R. & BAUM, M.B. (1988). Does chemotherapy improve survival in advanced breast cancer? A statistical overview. *Br. J. Cancer*, **57**, 615-618.

BERKSON, J. & GAGE, R.P. (1950). Calculation of survival rates for cancer. *Proc. Staff Meet. Mayo Clin.*, **25**, 270–286.

BLACK, W.C. & WELCH, G. (1993). Advances in diagnostic imaging and overestimation of disease prevalence and the benefits of therapy. *N. Engl. J. Med.*, **328**, 1237–1243.

CLARK, R.M., MCCULLOCH, P.B., LEVINE, M.N., LIPA, M., WILKINSON, R.H., MAHONEY, L.J., BASRUR, V.R., NAIR, B.D., MCDERMOT, R.S., WONG, C.S. & CORBETT, P.J. (1992). Randomized clinical trial to assess the effectiveness of breast irradiation following lumpectomy and axillary dissection for node-negative breast cancer. *J. Natl Cancer Inst.*, **84**, 683–689.

COEBERGH, J.W.W., VERHAGEN-TEULINGS, M.Th., CROMMELIN, M.A., MASSELING, E., VAN DER HEIJDEN, L.H. (1992). Netherlands, Eindhoven. In *Cancer Incidence in Five Continents*, Vol. VI. IARC Scientific Publications No. 120, Parkin, D.M., Muir, C.S., Whelan, S.L., Gao, Y.T., Ferlay, J. & Powell, J. (eds) pp. 666–669. International Agency of Research on Cancer: Lyon.

DANFORTH, D.N., FINDLAY, P.A., MCDONALD, H.D., LIPPMAN, M.E., REICHERT, C.M., D'ANGELO, T., GORREL, C.R., GERBER, N.L., LICHTER, A.S., ROSENBERG, S.A. & DEMOSS, E.S. (1986). Complete axillary lymph node dissection for stage I–II carcinoma of the breast. *J. Clin. Oncol.*, **4**, 655–662.

EARLY BREAST CANCER TRIALIST'S COLLABORATIVE GROUP (1992). Systemic treatment of early breasts cancer by hormonal, cytotoxic, or immune therapy. 133 randomized trials involving 31,000 recurrences and 24,000 deaths among 75,000 women. *Lancet*, **339**, 1–15, 71–85.

EWERTZ, M. (1993). Breast. In *Survival of Danish Cancer Patients 1953–1987*. Carstensen, B., Storm, H.H. & Schou, G. (eds). *APMIS*, **101** (Suppl. 33), 99–106.

FEINSTEIN, A.R., SOSIN, D.M. & WELLS, C.K. (1985). The Will Rogers Phenomenon. Stage migration and new diagnostic techniques as a source of misleading statistics for survival in cancer. *N. Engl. J. Med.*, **312**, 1604–1608.

FISHER, B., REDMOND, C., POISSON, R., MARGOLESE, R., WOLMARK, N., WICKERHAM, L., FISHER, E., DEUTSCH, M., CAPLAN, R., PILCH, Y., GLASS, A., SHIBATA, H., LERNER, H., TERZ, J. & SIDOROVICH, L. (1989). Eight-year results of a randomized clinical trial comparing total mastectomy and lumpectomy with or without irradiation in the treatment of breast cancer. *N. Engl J. Med.*, **320**, 822–828.

HAKULINEN, T. (1982). Cancer survival corrected for heterogeneity in patient withdrawal. *Biometrics*, **38**, 933–942.

HAKULINEN, T. & TENKANEN, L. (1987). Regression analysis of relative survival rates. *Appl. Stat.*, **36**, 309–317.

HAKULINEN, T., PUKKALA, E., HAKAMA, M., LEHTONEN, M., SAXEN, E. & TEPPO, L. (1981). Survival of cancer patients in Finland 1953–1974. *Ann. Clin. Res.*, **13** (Suppl. 31), 48–50.

HERMANEK, P. & SOBIN, L.N. (eds, 1987). Breast tumours. In *TNM Classification of Malignant Tumours. International Union Against Cancer*, Springer: Berlin.

JOENSUU, H. & TOIKKANEN, S. (1991). Comparison of breast carcinomas diagnosed in the 1980s with those diagnosed in the 1940s to 1960s. *Br. Med. J.*, **303**, 155–158.

LEVI, F., RANDIMBISON, L., VAN-CONG, T., FRANCESCHI, S. & LA VECCHIA, C. (1992). Trends in cancer survival in Vaud, Switzerland. *Eur. J. Cancer*, **28A**, 1490–1495.

MILLER, B.A., FEUER, E.J. & HANKEY, B.J. (1993). Recent incidence trends for breast cancer in women and the relevance of early detection: an update. *Ca. Cancer J. Clin.*, **43**, 27–41.

NAB, H.W., VOOGD, A.C., CROMMELIN, M.A., KLUCK, H.M., VAN DER HEIJDEN, L.H. & COEBERGH, J.W.W. (1993). Breast cancer in south-eastern Netherlands, 1960–1989: trends in incidence and mortality. *Eur. J. Cancer*, **29A**, 1557–1559.

SANT, M., GATTA, G., MICHELI, A., VERDECCHIA, A., CAPOCACCIA, R., CROSIGNANI, P. & BERRINO, F. (1991). Survival and age at diagnosis of breast cancer in a population-based cancer registry. *Eur. J. Cancer*, **27**, 981–984.